# GLD-SAIPAR Covid-19 survey in Zambia dataset

**DOI:** 10.1016/j.dib.2023.109735

**Published:** 2023-10-26

**Authors:** Erica Ann Metheney, Ellen Lust

**Affiliations:** University of Gothenburg, 405 30 Göteborg, Sweden

**Keywords:** Zambia, Covid, Authority, Governance, Panel, Survey

## Abstract

In March 2020, Zambia reported their first cases of Covid-19, with the government restricting non-essential travel, banning large public gatherings, and establishing emergency committees to spearhead efforts to contain the pandemic at a national level. The Governance and Local Development Institute (GLD) in collaboration with South African Institute for Policy and Research (SAIPAR) implemented a survey in 2020 to generate a dataset to provide better understanding of citizens' responses to such measures, and the impact of the pandemic on their individual lives and communities. To generate as complete a picture as possible of the local situations over time, we conducted two survey rounds. These rounds resulted in a total of 3925 completed surveys (1022 respondents took both rounds), gathering information on issues including knowledge of Covid-19; attitudes and fears surrounding health and economic impacts; social, economic, and health vulnerabilities; social distancing practices and other preventative measures. Particular attention was paid to the local variation in concerns over the social stigma, levels of enforcement, and engagement of different authorities.

Specifications Table

**Table:** 

Subject	Social Science
Specific subject area	Citizens’ experience and reactions to public health crises and government interventions.
Type of data	Tables (summary statistics), Survey Data (.rds,.dta,.tab)
How the data were acquired	The survey was administered via telephone by Ubuntu Research and Rural Development Company Ltd. enumerators. Responses were recorded using the SurveyToGo app from Dooblo on a tablet or computer. The full survey questionnaires (including questions excluded from the publicly available dataset) are available as supplemental files in English, Bemba, and Nyanja.
Data format	Raw
Description of data collection	The sampling frame consists of respondents to the 2019 Local Governance Performance Index (LGPI) survey [1] who consented to follow up contact and provided a phone number. A detailed description of the sampling plan of the 2019 LGPI survey can be found in the supplemental materials. Trained enumerators from Ubuntu Research and Rural Development Company Ltd. contacted respondents via phone and recorded responses in the SurveyToGo app. Enumerators were trained to first try to contact the previous LGPI respondent, but if that was not possible, a replacement respondent was allowed. Therefore, the sampling frame from Round 2 consisted of the Round 1 sampling frame, minus those who refused participation or follow up contact, and replacement respondents who agreed to follow up contact. Fielding for Round 1 took place from July 2, 2020 to August 13, 2020 and for Round 2 from November 26, 2020 to January 22, 2021.
Data source location	Country: Zambia Provinces: Central, Copper Belt, Eastern, Luapula, Lusaka, Muchinga, North-Western, Northern, Southern, Western
Data accessibility	The survey data and survey questionnaires are available via the Harvard Dataverse. Repository name: Harvard Dataverse Data identification number: 10.7910/DVN/JIKM9F Direct URL to data: https://doi.org/10.7910/DVN/JIKM9F

## 1 Value of the Data

• These data are useful because they provide information on issues including knowledge of Covid-19; attitudes and fears surrounding health and economic impacts; social, economic, and health vulnerabilities; social distancing practices and other preventative measures. The data also contains comprehensive information regarding the local variation in concerns over the social stigma, levels of enforcement, and engagement of different authorities.
• The data can be used by government officials to better understand citizen reactions to government measures; by CSOs and donors to understand the needs during healthcare crises, and by citizens in Zambia to gain a broader understanding of events and experiences during the pandemic.
• These data are provided as single merged file so that the data can be used to examine how individual and community experiences changed during the Covid-19 pandemic. When merged with the LGPI 2019 or Zambia Election Panel Survey (ZEPS) [2] surveys, the data can also be used to examine the relationship between pre- and during pandemic conditions and the impact of the pandemic on elections respectively. They also provide a foundation to build future experiments regarding crises.

## 2 Objective

In Fall 2019, GLD administered the Local Governance Performance Index survey (LGPI 2019) in two large regions of Zambia. This survey provided a comprehensive overview of individuals and their communities just prior to the Covid-19 pandemic. Moreover, the survey had generated a bank of phone numbers that could be used to contact respondents safely during Covid. Given the large number of LGPI 2019 respondents who consented to follow up contact and provided telephone numbers to do so, we sought out to generate this panel dataset to:

1. Generate information and actionable insights for government officials, CSOs, donors, other stakeholders, and the public
2. Provide insights for academics and practitioners concerned with understanding the relationships between individual, household, and community vulnerabilities and resilience in the face of crises.
3. Obtain data collected during the Covid-19 pandemic that can be compared to data collected just prior to the pandemic.

Additionally, this data article will provide more context to the article *The ABCs of Covid-19 prevention in Malawi: Authority, benefits, and costs of compliance* [3] that analyzed a conjoint experiment (not included in this dataset) contained within the Zambia Covid Panel Survey by providing full access to the survey data that surrounded the experiment.

## 3 Data Description

The data was collected in two rounds; early in the Covid-19 pandemic, from July 2, 2020–August 13, 2020 (R1: *n* = 2142) and mid-pandemic, from November 26, 2020–January 22, 2021 (R2: *n* = 1783). The data codebook, available as a supplemental file in the Harvard Dataverse repository, contains information on variable names, question wording, and frequency of responses. Not all questions or topics were necessary or appropriate for both rounds. Table 1 charts the different topics investigated in each round of the survey. The data is provided as a single dataset with Round 1 and Round 2 merged such that all responses in the same row came from the same individual. All variable names indicate if the variable came from the Round 1 or Round 2 survey.

**Table 1:** Summary of topics covered by each survey round.

Topic	Round 1	Round 2
Respondent Demographics	x	x
Knowledge of Covid-19	x	
Sources of Covid-19 Information	x	
Attitudes Towards Covid-19 Testing	x	
Precautionary Behaviors	x	x
Use of Healthcare	x	x
Community Restrictions	x	x
Economic Impacts and Fears	x	x
Hunger Impact and Fears	x	x
Health Impact and Fears	x	x
Safety Impact and Fears	x	x
Social Assistance and Relief	x	x
Perceptions of Government	x	x

Respondents were contacted by phone in all Zambian provinces. However, due to the sampling of the Local Governance Performance Index (LGPI) 2019 dataset, respondents were drawn disproportionately from Central, Eastern, Lusaka, and Muchinga provinces. This survey is therefore not nationally representative. A demographic breakdown of the respondents’ gender, location, and age is shown for all survey rounds in Table 2.

**Table 2:** Respondent characteristics, by survey round.

	R1	R2
**Total**	2142	1783
***Gender***		
Men	49%	51%
Women	51%	49%
***Province***		
Central	8%	7%
Copper Belt	2%	2%
Eastern	24%	25%
Luapula	<1%	<1%
Lusaka	47%	46%
Muchinga	14%	14%
North-Western	<1%	<1%
Northern	4%	4%
Southern	<1%	1%
Western	<1%	<1%
DK/RA Region	0	0
***Age***		
18–25	19%	17%
26–45	55%	58%
46–65	21%	21%
Older than 65	5%	4%

Due to data privacy requirements, in order to merge the dataset with the LGPI 2019 Household survey, a formal request should be made by contacting the Governance and Local Development Institute directly at data@gld.gu.se. To merge the data with the Zambia Election Panel Surveys (ZEPS), use the ZEPS-ZCS matching keys which can be found in the ZEPS dataset on Harvard Dataverse [4].

## 4 Experimental Design, Materials and Methods

This dataset is the result of a collaborative project by an international group of experts on democracy, governance, and survey methodology in Zambia and beyond. The surveys include multiple choice, rating scale, and open-ended questions. In certain parts of the survey, a question or set of questions is asked in loops for a list of responses to a previous question. The instruments also included survey experiments and a small number of questions that are not included in the released data by request of project collaborators. For full transparency around the flow of the survey, we provide the full questionnaire, with removed questions clearly marked.

The survey was coded using SurveyToGo Studio software from Dooblo. Logic patterns were utilized to decrease survey time and to create a comfortable flow through the survey. The survey questionnaire was originally written in English and then was translated into Bemba and Nyanja. The translated versions are available in the supplemental materials.

The surveys were implemented by a team of enumerators from the Ubuntu Research and the Rural Development Company Ltd. using the freely available SurveyToGo app. Enumerators received training in research ethics, effective administration of surveys, sampling procedures, and use of the SurveyToGo app. The survey was administered via telephone in two rounds.

The sampling frame for the first round of the panel consisted of 4280 contacts from individuals who:

1. Took the Local Governance Performance Index (LGPI) 2019 survey,
2. Gave consent for follow-up contact, and
3. Provided a telephone number

Enumerators were trained to first attempt to contact the previous LGPI 2019 respondent. If it was not possible to contact the previous respondent, the enumerator then offered the survey to the individual who answered the phone. The sampling frame for the second round of the panel consisted of 4072 contacts from individuals who

1. Were part of the Round 1 sampling frame but were not contacted
2. Took the Round 1 survey and consented to follow up contact

## Ethics Statements

This study received research ethics and regulatory approval from the University of Zambia (970-2020). We note that the Swedish Ethnical Review Authority will not provide IRB approval for research conducted outside of Sweden.

This study was conducted during the Covid-19 pandemic, and as a result three measures were taken to reduce face-to-face interactions: 1) the survey was administered via telephone, 2) enumerator training was hosted online, and 3) enumerators were able to administer surveys from home.

Informed consent was received for each survey in accordance with General Data Protection Regulations (GDPR) requirements. Only respondents older than 18 years old were allowed to participate. Additionally, participants were financially compensated for the cost of the telephone call. The consent statement was as follows:

We are currently conducting a survey on Covid-19 and would like to talk to you today. Participation is voluntary, and your answers will be confidential. They will be put together with about 3500 other people we are talking to, to get an overall picture. It will be impossible to pick you out from what you say, so please feel free to tell us what you think. This interview will take about 30 min.

There is no penalty for refusing to participate. We would like your opinion with the knowledge that there are no right or wrong answers to these questions and that you may ask for clarification or stop the survey at any time. You are also free to skip questions you consider personal or invasive without penalty.

We are able to offer you 20 Kwacha in airtime for completing the survey. 10 Kwacha will be credited immediately after the survey and the remaining 10 Kwacha will be credited 1 month later due to administrative restrictions.

If you have any questions, please contact: Marja HinfelaarSAIPAR, 22 Busuma Rd, Sunningdale, Lusaka +260 977751317 marja.hinfelaar@saipar.org UNZABRECP.O. Box 50110, Ridgeway Campus, Lusaka+260-1-256067 unzarec@unza.zm

Are you willing to participate in this survey, either now or at another time?

## CRediT authorship contribution statement

**Erica Ann Metheney:** Data curation, Writing – original draft. **Ellen Lust:** Writing – review & editing.

## Data Availability

GLD-SAIPAR Covid-19 Survey in Zambia (Original data) (Dataverse)
